# Advances in CRISPR/Cas gene therapy for inborn errors of immunity

**DOI:** 10.3389/fimmu.2023.1111777

**Published:** 2023-03-27

**Authors:** Xinyi Liu, Guanglei Li, Yin Liu, Fuling Zhou, Xingxu Huang, Kui Li

**Affiliations:** ^1^ Shenzhen Branch, Guangdong Laboratory for Lingnan Modern Agriculture, Agricultural Genomics Institute at Shenzhen, Chinese Academy of Agricultural Sciences, Shenzhen, China; ^2^ School of Life Science and Technology, ShanghaiTech University, Shanghai, China; ^3^ Department of Hematology, Zhongnan Hospital of Wuhan University, Wuhan University, Wuhan, China

**Keywords:** inborn errors of immunity (IEIs), Primary Immunodeficiencies (PIDs), CRISPR/Cas, gene therapy, gene editing, base editing, prime editing

## Abstract

Inborn errors of immunity (IEIs) are a group of inherited disorders caused by mutations in the protein-coding genes involved in innate and/or adaptive immunity. Hematopoietic stem cell transplantation (HSCT) is a mainstay definitive therapy for many severe IEIs. However, the lack of HLA-matched donors increases the risk of developing severe immunological complications. Gene therapy provides long-term clinical benefits and could be an attractive therapeutic strategy for IEIs. In this review, we describe the development and evolution of clustered regularly interspaced short palindromic repeat (CRISPR)/CRISPR-associated proteins (Cas) gene-editing systems, including double-strand break (DSB)-based gene editing and DSB-free base editing or prime editing systems. Here, we discuss the advances in and issues associated with CRISPR/Cas gene editing tools and their potential as therapeutic alternatives for IEIs. We also highlight the progress of preclinical studies for the treatment of human genetic diseases, including IEIs, using CRISR/Cas and ongoing clinical trials based on this versatile technology.

## 1 Introduction

Inborn errors of immunity (IEIs), also known as primary immunodeficiencies (PIDs), are a group of inherited disorders caused by monogenic mutations in encoded proteins involved in immune responses. IEIs have been considered rare diseases with an overall estimated prevalence of approximately 1/10,000 to 1/50,000; however, along with the continued discovery of novel IEIs, the prevalence of these diseases appears to have been underestimated by 10-fold, with IEIs actually affecting more than 1 in 1000 to 1 in 5000 births (1). The advent and application of next-generation sequencing has expedited the discovery of novel gene defects that affect the development and function of the innate or adaptive immune system (2). Currently, 430 distinct immunological disorders caused by known genetic defects have been identified, and they have diverse underlying manifestations, such as susceptibility to infections, allergy, autoimmunity, autoinflammation, and malignancy (1, 3).

Patients bearing a disease-causing mutation that disables the immune system usually suffering from life-threatening infectious disease, early diagnosis and proper management are of paramount importance to prevent the development of complications, especially for those with severe complications. The development of high throughput sequencing technologies and advances in diagnostic methods have resulted in a growing number of IEIs could be identified in pediatric age (4). The treatment options for IEIs include small-molecule inhibitor administration, immunoglobulin/enzyme replacement therapy, hematopoietic stem cell transplantation (HSCT), and gene therapy. These treatments have developed rapidly based on remarkable biotechnological advances capable of characterizing their molecular mechanisms (5). Small molecule inhibitors like ruxolitinib, a Janus kinase (JAK) family protein tyrosine kinase inhibitor, has been successfully applied in treatment of infections caused by gain of function (GOF) mutations of signal transducer and activator of transcription (STAT1) (6, 7). Intravenous or subcutaneous injection of immunoglobulin improves the immune phenotype of patients with absence of B cells or deficient antibody production (8–10). Enzyme replacement therapy (ERT) with the form of polyethylene-glycol-modified ADA (PEG-ADA) administration displayed significant clinical improvement in adenosine deaminase-deficient severe combined immunodeficiency (ADA-SCID) patients (11–13). Targeted drugs ameliorate the clinical symptoms of an IEI, however, the effects are often poorly sustained and substantial treatment burdens are heavy over life-long medication. Recently, HSCT has been the mainstay definitive therapy for severe combined immunodeficiency (SCID) and other severe forms of IEIs. HSCT from a well-matched donor can lead to a lifelong cure for more than 90% of SCID patients, who subsequently present very robust and long-lasting immune reconstitution (14, 15). However, the availability of HLA-matched donors is limited for a significant number of patients. Moreover, HSCT using an HLA-mismatched donor increases the risk of developing severe immunological complications, such as graft-versus-host disease (GvHD) and infections (16–18). Considering the pathogenic mutations of coding genes, gene therapy can provide long-term clinical benefits and thus represents an attractive therapeutic strategy.

Gene therapy for IEIs has been developed to provide an autologous HSCT option by adding a normal copy of the responsible disease-related gene or correcting the mutation in the patient’s own HSCs. For loss-of-function mutations, viral vector-mediated gene augmentation, which provides the correct copy of defective genes, can be used for gene therapy. Gamma retroviral vectors were used as delivery vectors in early clinical trials for gene therapy of IEIs, including ADA-SCID, X-linked SCID (X-SCID), Wiskott–Aldrich syndrome (WAS), and X-linked chronic granulomatous disease (X-CGD) (19–27). To prevent insertional oncogenic mutagenesis, self-inactivating (SIN) lentiviral vectors have been developed and become the most commonly used vectors for insertion of therapeutic genes into hematopoietic stem and progenitor cells (HSPCs) in both research and clinical trials for some IEIs, including X-SCID, ADA-SCID, X-CGD, and WAS (18, 28, 29). For gain-of-function (GOF) mutations or genes that require precise regulation, *in situ* correction of the pathogenic mutations is needed and gene editing systems provide useful tools to revise GOF mutations. Here, we describe advances in precise genome editing using the lustered regularly interspaced short palindromic repeat (CRISPR)/CRISPR-associated proteins (Cas) gene editing system and reveal the great potential of CRISPR/Cas-based gene therapy for the treatment of IEIs. In this review, we focus on DNA editing using CRISPR/Cas; thus, RNA editing will not be included.

## 2 Gene editing systems mediate efficient and precise genome editing

**Figure 1:**
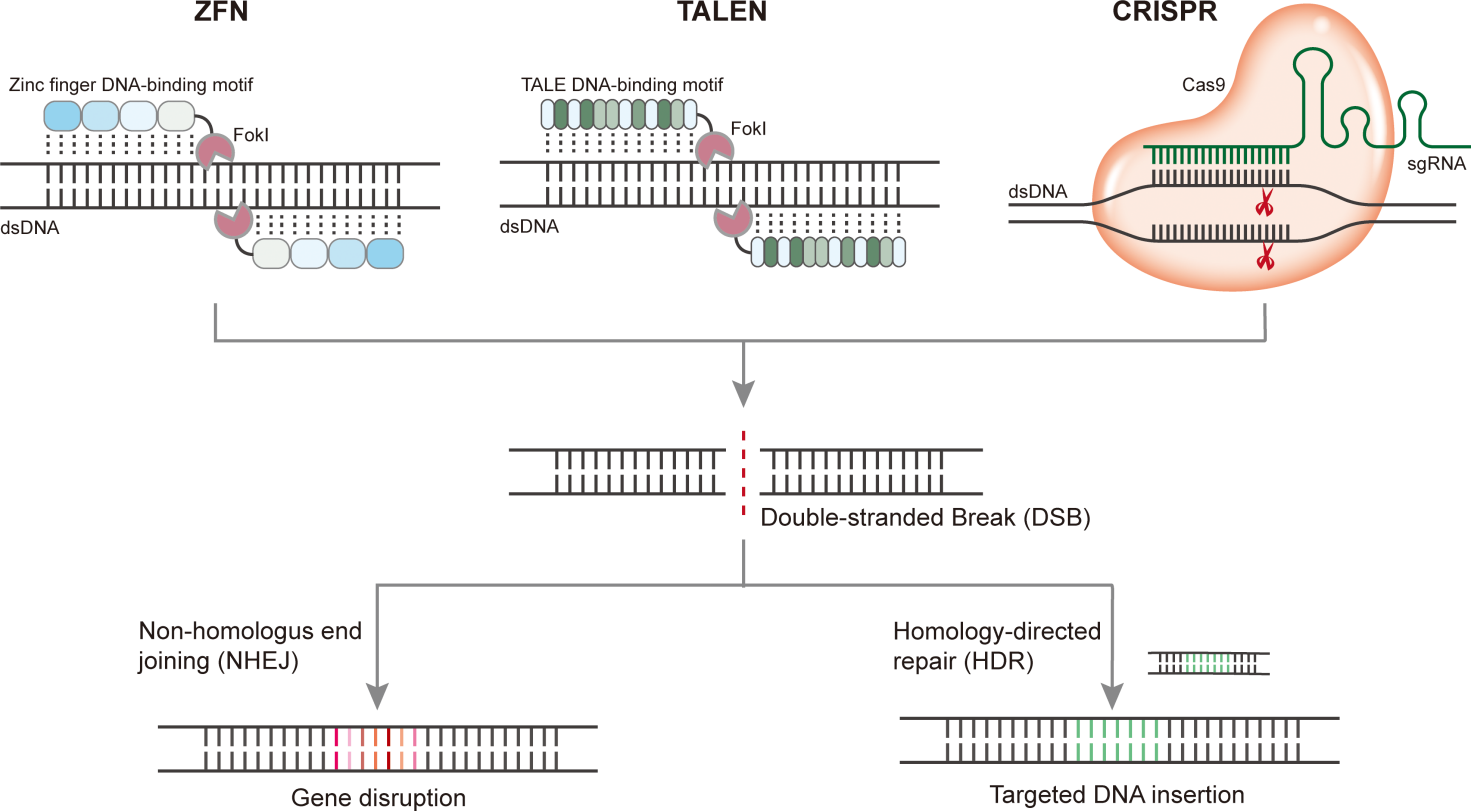
Programmable gene editing using ZFN, TALEN, and CRISPR. Upon cleavage of target DNA, DSBs induced by nuclease can be repaired by the error-prone nonhomologous end joining (NHEJ) pathway which often resulting in nucleotide insertions or deletions (indels) thus disturb the target gene, or by homology-directed repair (HDR) pathway which enables gene correction or insertion of a transgene when a homologous donor DNA template are provided.

Targeted genome editing introduces stable genetic modifications at sites of interest using engineered nucleases. Methods for eukaryotic genomic manipulation have evolved rapidly over the past decade. Programmable nucleases, including zinc-finger nucleases (ZFNs), transcription activator-like effector nucleases (TALENs), and CRISPR/Cas, are three popular nucleases that have been engineered to induce double-strand breaks (DSBs) at specific endogenous gene loci to enable targeted genome manipulation. DSBs can be repaired by the error-prone non-homologous end joining (NHEJ) pathway, which often results in nucleotide insertions or deletions (indels), thus inactivating the gene, or by the homology-directed repair (HDR) pathway, which enables gene correction or insertion of a transgene when a homologous donor DNA template is provided (**Figure 1**). Compared with ZFNs and TALENs, which recognize target sequences through protein-DNA interactions, CRISPR/Cas genome editing systems are RNA-guided nuclease target sequences based on RNA-DNA base pairing. For each new target site, ZFN and TALEN require the engineering of a new protein, whereas the CRISPR/Cas system can be reprogrammed simply by RNA redesign, thus offering distinct advantages.

### 2.1 Gene editing based on DSBs induced by CRISPR/Cas

CRISPR/Cas systems function as adaptive immune systems in bacteria and archaea and have been exploited for biological research and translational applications. The widely used CRISPR/Cas9 and CRISPR/Cas12 systems belong to the class 2 CRISPR/Cas system, which employs single large effector proteins with multiple domains for nucleic acid cleavage (30). In the CRISPR/Cas9 system, target DNA is cleaved by the formation of ribonucleoprotein complexes with CRISPR RNAs (crRNAs) and trans-activating crRNAs (tracrRNAs) in their native context, whereas in genome editing applications, the tracrRNA and crRNA complexes have been engineered as single guide RNA (sgRNA) chimeras to facilitate simple and versatile genome manipulation (31). Two distinct nuclease domains of Cas9, the HNH nuclease domain and RuvC-like nuclease domain, are responsible for the cleavage of the guide RNA–bound target DNA strand and the protospacer adjacent motif (PAM)-containing non-target DNA strand, respectively (31, 32). Inactivation of either domain abolishes the nuclease activity of Cas9 and generates a nickase, while the simultaneous inactivation of both domains results in a catalytically dead Cas9 (dCas9) (3). Cas9 nickase (nCas9) has been applied to improve the targeting precision of Cas9 by using paired nickases together with two sgRNAs (33, 34), and precision genome editing tools have been developed, such as base editors (BEs) and prime editors (35–37). The sequence-specific DNA-binding property of dCas9 allows for transcriptional regulation and epigenetic modification without genetically altering the DNA sequence (38, 39) or chromosome imaging in live cells (40). Moreover, dCas9 was harnessed to increase the specificity of CRISPR/Cas9-mediated DSB formation by fusing to the catalytic domain of Fok1 (41, 42). The presence of a PAM sequence is critical for Cas protein binding, and a number of Cas9 protein orthologs have been discovered to expand targeting scope. For example, SaCas9 recognizes the NNGRRT PAM sequence (43), CjCas9 recognizes the NNNNACA PAM sequence (44), NmCas9 recognizes the NNNNGATT PAM sequence (45), and ScCas9 recognizes the NGN PAM sequence (46). Compared with Cas9, Cas12 possesses a single RuvC-like domain that cleaves both the guide RNA complementary DNA strand and the noncomplementary DNA strand (30). The CRISPR/Cas12 system encompasses several sub-type effectors, many of which are guided by a single crRNA, although some (such as Cas12b, Cas12c2, and Cas12e) require both crRNA and tracrRNA for activation (47, 48). Cas12a (formerly Cpf1) and Cas12b (formerly C2c1) are two Cas12 effectors that have been studied in detail. Cas12a is a single RNA-guided endonuclease that recognizes a T-rich PAM sequence and mediates robust DNA interference *via* a single RuvC catalytic domain, and it has been widely used for genome editing applications (49). Compared with Cas12a, Cas12b requires both tracrRNA and crRNA for DNA cleavage. Guided by chimeric sgRNA, engineered Cas12b facilitates robust genome editing in human cells and mice with high specificity (50, 51). Recently, miniature Cas12f has been revealed as a compact nuclease with high efficiency that offers more options for therapeutic applications (52–54). For several Cas12 variants, RNA-guided target recognition and binding unleash their robust, indiscriminate single-stranded DNA or RNA cleavage activity (55, 56). This collateral cleavage activity has facilitated the development of numerous strategies for rapid nucleic acid detection and portable diagnosis (55, 57, 58).

**Figure 2:**
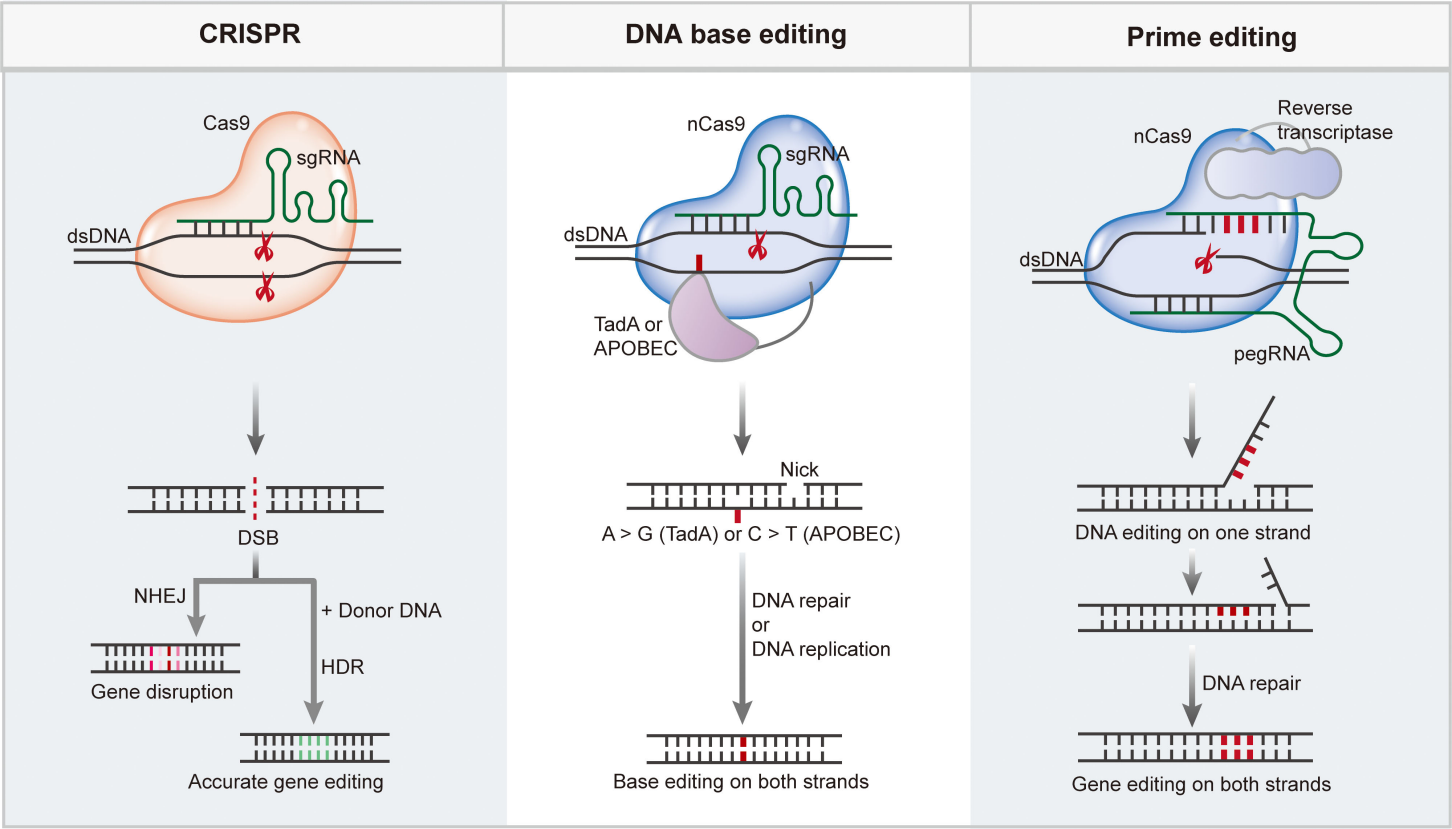
CRISPR/Cas mediate efficient and precise genome editing. CRISPR/Cas nucleases generate DSBs upon target recognition, DSBs can be repaired through NHEJ pathway to disturb the target gene, or through HDR pathway to enable targeted gene insertion or replacement. Base editing is performed by a fusion complex of catalytically impaired Cas nuclease and a single strand DNA (ssDNA) deaminase enzyme. Base editing enabling efficient and precisely targeted base conversion at single base resolution without producing DSBs or requiring a donor DNA template. Prime editing requires a complex of Cas9 H840A nickase fused to an engineered reverse transcriptase enzyme as well as a pegRNA, enables base conversions, insertions or deletions in a precise way.

Upon nuclease cleavage, two main DNA repair pathways, NHEJ and HDR, are involved in the repair of nuclease-induced DSBs (**Figure 2**, left panel). In most mammalian cells, error-prone NHEJ is the predominant repair pathway, which often introduces indels at sites of DSBs, resulting in the disruption of target gene sequences or regulatory elements (59, 60). In some cases, indels resulting from NHEJ are not sufficient to disrupt the function of gene clusters or regulatory sequences; thus, large deletions are more desirable. Using dual guide RNAs targeting adjacent regions of a chromosome sequence, Cas9 nuclease can introduce two DSBs simultaneously, which often leads to the deletion or inversion of the intervening sequence (61, 62). By the delivery of Cas9 nuclease coupled with paired sgRNAs flanking the mutated Dmd exon23, efficient target sequence excision, and phenotypic restoration were achieved in the mdx mouse model of Duchenne muscular dystrophy (DMD) (63). CRISPR/Cas-mediated NHEJ exhibits great potential for the correction of dominant-negative mutations underlying diseases. Delivery of the CRISPR/Cas9:sgRNA complex through cationic lipids into a mouse model of human genetic deafness disrupted the dominant inherited mutation in *Tmc1* and ameliorated the pathogenic phenotype (64). The NHEJ repair process is fast and flexible, and frameshifts resulting from indels are likely to generate premature termination codons, which might lead to nonsense-mediated decay of mRNA and prevent protein translation (65). Disruption of multiple genes simultaneously could be achieved by simultaneously targeting multiple genomic loci (66, 67). Although NHEJ mediates efficient gene disruption, the editing results are usually uncontrollable due to the formation of indels at sites of DSBs, which has hindered its broad application in clinical transformation.

In the presence of a homologous donor template, the HDR repair pathway can be initiated to insert a fragment of interest through homologous recombination at a precisely targeted genomic location. The HDR pathway competes with NHEJ for the repair of nuclease-induced DSBs, and selection of a repair pathway is influenced by many factors, such as the structure and sequence context of cutting ends, the format of the donor template, the state of the cell cycle, etc. (65). The donor template for HDR could be a double-stranded DNA (dsDNA)-like plasmid donor or single-stranded DNA (ssDNA), such as ssODN. ssDNA usually mediates a more efficient HDR process, whereas dsDNA allows for larger DNA fragment knock-in (68). With a proper donor, the repair of CRISPR/Cas-induced DSBs by HDR can introduce a variety of genome edits, including gene insertion or replacement (69, 70). Compared with the NHEJ process, which is efficient in most mammalian cells, HDR is inefficient and generally active only in dividing cells; moreover, the frequency of intended sequence insertion can be improved by synchronizing cells at the S and G2 phases using chemical factors (71). Efforts to improve the efficiency of HDR include modifying the length and symmetry of homologous arms, inhibiting the NHEJ pathway, or enhancing the HDR pathway by manipulating core factors (72, 73).

CRISPR/Cas nuclease-mediated gene disruption is widely used in biomedical research. Efficient and specific abrogation of protein function is of particular interest in the study and treatment of genetic disorders caused by dominant-negative mutations. HDR can be used to either insert a gene fragment into a specific site or replace defective genes in situ. However, CRISPR/Cas9-induced DSBs at a target locus often result in undesired mutations and HDR appears to be inefficient in quiescent cell types. Moreover, more than half of the known human pathogenic mutations are point mutations (74); thus, alternative approaches that efficiently and precisely install or reverse pathogenic mutations are highly desirable.

### 2.2 Gene editing based on DNA base editing systems

DNA BEs are mainly composed of a catalytically impaired Cas nuclease and ssDNA deaminase enzyme. They enable efficient and precise targeted base conversions at a single-base resolution without producing DSBs or requiring a donor DNA template (74). DNA BEs have been categorized into two major classes: cytosine BEs (CBEs) and adenine BEs (ABEs), of which CBEs mediate C-G to T-A base conversions using naturally existing cytidine deaminases while ABEs mediate A-T to G-C base conversions through revolutionized *Escherichia coli* tRNA adenosine deaminase TadA (**Figure 2**, middle panel) (35, 36).

#### 2.2.1 Development of DNA BEs

CBEs were first reported by Liu and co-workers in 2016 (35), and base editor 1 (BE1) was developed by fusing a naturally existed cytidine deaminase rAPOBEC1 to dCas9 (Cas9 variants containing both D10A and H840A mutations). rAPOBEC1 catalyzes cytosine (C) deamination into uracil (U), which is recognized as thymine (T) in base-pairing process; this results in the conversion of a C-G base pair to T-A base pair. To elevate base editing efficiencies, uracil DNA glycosylase inhibitor (UGI) was introduced into BE2 to inhibit uracil N-glycosylase (UNG), which mediates base excision repair (BER) of U-G mismatch, usually resulting in the reversion of the U-G intermediate back to a C-G base pair. BE3 was generated by replacing dCas9 of BE2 with an nCas9 (D10A mutation), which specifically nicks the non-deaminated strand to induce mismatch repair (MMR) process resulting in U-G mismatch transformation into the desired T-A base-pair. BE3 is the first reported CBE that enabled efficient C-G to T-A base conversions in mammalian cells with an editing window at positions 4–8 (counting the first nucleotide of the protospacer as position 1 with the PAM at positions 21–23); a small frequency of indels was also observed (35). Similar to cytosine that deaminates into uracil, deamination of adenosine (A) yields inosine, which pairs with C and therefore is recognized as guanine (G). To achieve A-T to G-C base conversion, Liu’s group performed protein evolution and engineering of an adenosine deaminase TadA due to the lack of any known natural enzymes capable of acting on ssDNA (36). Following several rounds of evolution, a TadA mutation (TadA*) was identified and fused to nCas9 in a heterodimeric form (wtTadA-TadA* complex) to generate ABE7.10. ABE7.10 enables efficient A-T to G-C base conversion in mammalian cells with very high product purity and very low rates of indels within an active editing window of positions 4–7 (counting the first nucleotide of the protospacer as position 1 with the PAM at positions 21–23) (36).

Both CBE and ABE are powerful tools for irreversible DNA base transitions (A-G, C-T, G-A, and T-C) in various cell types. The editing efficiency of BEs was continuously increased through linker selection, additional UGI appending, nuclear localization sequence modification, codon optimization, and Cas protein or deaminase engineering (75–78). For example, BE4max and AncBE4max were developed by optimization of the nuclear localization sequence and codon usage, and ancestral reconstruction of the deaminase, respectively (76). Joung and co-workers developed miniABEmax by removing wild type TadA monomer and demonstrated that this component is unnecessary for ABE activity (77). Further, ABEs were considered to have poor compatibility with some Cas homologs (such as Cas12). In contrast to CBEs, the developed ABE8e through deaminase evolution exhibits substantially improved editing efficiencies especially when paired with a variety of Cas homologs (78). The invention of CBEs and ABEs has enabled efficient base transitions (A-G, C-T, G-A, and T-C). Despite being valuable, the utility of these two category BEs in correcting pathogenic variants is hindered due to the inability of base transversions. To achieve C to G base transversion in mammalian cells, cytosine deaminase rAPOBEC1 and uracil excision-related protein UNG were fused with nCas9 by researchers (79, 80). Another group developed CGBE by the fusion of nCas9 with rAPOBEC1 and base excision repair protein rXRCC1 to enable efficient C-G base transversion in WCW, ACC, or GCT sequence contexts with a precise editing window (81). To further expand the editing competency of the system, dual-function BEs which enable concurrent cytosine and adenine editing were developed by simultaneously fusing cytosine and adenine deaminases into Cas9 variants and applied in mammalian cells and plants (82–87). Most recently, an adenine transversion base editor, AYBE, was developed for A-C and A-T transversion editing in mammalian cells by fusing an ABE with hypoxanthine excision protein N-methylpurine DNA glycosylase (MPG) (88).

#### 2.2.2 Improvements made for precise base editing

The improvements of BEs for precise base editing have mainly focused on several aspects, such as broadening the targeting scope, enhancing product purity, reducing off-target activity, and others. As mentioned above, BEs employ Cas proteins to localize deaminases to the target regions, and target bases must be positioned in the editing window to ensure efficient base editing. Thus, the PAM availability together with the width of the editing window determines the targeting scope of base editing. SpCas9 requires an NGG PAM for target DNA recognition and binding; this PAM requirement restricts the target space of SpCas9 to every 8 bp on average in the human genome (66). To broaden the targeting scope of BEs, other Cas orthologs and engineered Cas variants recognizing different PAMs have been incorporated into BEs (75, 89–94). Among Cas orthologs, SaCas9 and ScCas9 are two smaller Cas9 proteins, and BEs developed with these homologs should be easier to deliver *in vivo* (75, 93). Engineered SpCas9 variants VQR-Cas9, EQR-Cas9, VRER-Cas9, xCas9, SpCas9-NG, and Cas9-SpRY with alternative PAMs have been employed to develop BEs (89–92). In addition to pursuing PAM availability, altering the location and/or width of the editing window by replacing Cas and/or deaminase of BEs also expands the number of targetable sites. BEs with different Cas proteins or deaminases possess different editing windows. By combining pmCDA1 with nCas9, Nishida et al. developed a cytosine base editing system named ‘Target-AID’ that enabled targeted C-G to T-A base conversion in yeast and mammalian cells with an editing window shift comparable to that of BE3 (95). BEs with AID and A3A deaminases typically have wider windows, and CBEs containing highly active deaminases, such as human AID and some APOBEC3 family members, have been developed to mediate base conversions with high efficiency in different editing contexts (96, 97). The editing window of BEs generated with circularly permuted Cas9 expanded from ~4–5 nucleotides to ~8–9 nucleotides while also retaining product purity (98). Except for SpCas9 and engineered variants, the editing efficiency of ABE variants developed with other Cas enzymes is modest. The poor compatibility of ABEs has been overcome through the evolution of ABE8, which exhibits substantially improved editing efficiencies when paired with a variety of Cas homologs, including Cas12 enzymes (78). By combining the rapidly expanding Cas effectors with the growing set of deaminases, the targeting scope of BEs has been substantially expanded.

The product purity of base editing is a critical aspect that should be considered when optimizing BEs, especially for therapeutic applications. Ideal DNA BEs enable efficient and precise target-base conversion without generating excess by-products, unexpected edits, or detectable indels. Bystander editing occurs when multiple editable nucleotides are present within the editing window because the deaminase of BEs can deaminate all cytosines or adenines within the activity window. Due to degeneracy in the genetic code, some bystander edits will result in a synonymous mutation, whereas in some other cases, bystander editing results in amino acid alteration and is often problematic. Engineered deaminase variants with narrow activity windows or high sequence context dependence have been integrated into BEs to reduce or avoid bystander editing (77, 89, 97). The presence of unexpected edits caused by UNG-mediated uracil removing reduces the product purity of CBEs; additional expression or recruitment of UGI copies to the BE3 construct has been demonstrated to decrease unexpected editing and increase editing efficiency (75, 99, 100). Editing by BEs can also yield detectable indels due to the nicks formed on the deaminated strand by DNA-(apurinic or apyrimidinic site) lyase (AP lyase) and non-deaminated strand by Cas9 nickase; the two nicks may result in a DSB which is likely to be repaired by indel-prone NHEJ process. The Mu bacteriophage-derived Gam protein (Mu-GAM) binds to the free ends of DSBs and protects them from degradation (101), and fusing CBEs with Mu-GAM leads to substantial reduction of indels in human cells, rabbit embryos, and human tripronuclear (3PN) zygotes (75, 102, 103). Compared to CBEs, ABEs typically exhibit high product purity as well as very low and often undetectable indel frequencies, presumably because the removal of inosine is substantially inefficient and causes fewer nicks in the deaminated strand, resulting in fewer DSBs and indels (74).

DNA BE-induced off-target mutations have been reported (104–106), and genome-wide and transcriptome-wide off-target effects of BEs can occur in a Cas-dependent or Cas-independent manner. The former is usually caused by the Cas effector and sgRNA binding to sequences that are similar to the on-target locus, and the latter is due to the overexpression of deaminases that can randomly deaminate accessible nucleotides. To combat Cas-dependent off-target effects, engineered Cas9 variants with high fidelity, such as Cas9-HF and sniper-Cas9, have been incorporated into CBE structures (107, 108). SgRNA truncation and deaminase embedding have also been used to reduce the off-target editing of BEs (96, 109). Cas-independent DNA off-target as well as RNA off-target effects of CBEs can be ameliorated by engineering cytosine deaminase to generate various high-specific deaminase variants. For example, engineered rAPOBEC1 and human APOBEC3 family members have been incorporated into CBEs to reduce Cas-independent off-target effects (89, 110, 111). SECURE-BE3, another CBE variant using engineered rAPOBEC1 to deaminate cytosines, has been reported to reduce RNA off-target edits and narrow the editing window (77). Additional deaminases have also been identified and employed to generate next-generation CBEs with minimal Cas-independent DNA and RNA off-target edits (112). Although no genome-wide off-target A-T to G-C base editing has been observed, ABE can create substantial off-target edits on RNA (104, 105). Similar to CBEs, rational engineering of adenine deaminase can minimize the Cas9-independent RNA editing activity of ABEs (77, 111, 113, 114). Additionally, delivering BEs as purified ribonucleoprotein (RNP) complexes instead of DNA constructs to limit the exposure time of BEs greatly decreases off-target editing while maintaining comparable on-target editing (115).

#### 2.2.3 Base editing in inherited disorders

BEs utilize Cas effector-tethered nucleotide deaminases to induce efficient target base substitutions at a single-base resolution without introducing DSBs, which enables the use of base editing as a new therapeutic option for disorders caused by point mutations. Numerous studies have demonstrated the ability of BEs to correct pathogenic mutations underlying various human genetic diseases, including Duchenne muscular dystrophy (116), metabolic liver diseases (117, 118), hereditary deafness (64), progeria (23), Marfan syndrome (119), sickle cell disease (120), β-thalassemia (121), among others. Efficient modeling of pathogenic point mutations using BEs has also been performed in cultured human primary cells (122), embryos (123, 124), and various organisms, including mice (125), rabbits (102), pigs (126), and non-human primates (127–129). In addition to modeling or correcting human pathogenic point mutations, BEs can be used to regulate gene expression in a more precise manner. By targeting CAA, CAG, CGA, and TGG, CBEs can introduce premature stop codons, which usually result in the degradation of the target mRNA *via* nonsense-mediated decay (NMD), thus silencing gene expression (130, 131). Gene expression can also be shut down by ABE-mediated destruction of the start codon, ATG (132). Decreased abundance of abnormally expressed proteins could palliate the disease progress of these incurable disorders. In addition, modification of alternative splicing sites (donor or acceptor) using BEs facilitates selective skipping of mutation-containing exons while maintaining the preferred normal isoforms for gene therapy (133).

A major application of BEs is modeling or correcting point mutations underlying human genetic diseases along with the engineering of deaminase enzymes as well as Cas effectors. The editing characteristics of BEs, including the targeting scope, product purity, and potential off-target edits, have been improved to a large extent; thus, more BE variants with therapeutic potential are available.

### 2.3 Gene editing based on prime editing systems

Although BEs have been considered a safer and more precise genome editing tool, they can only mediate very limited editing types. Prime editors (PEs) have emerged as a genome-editing approach to enable all 12 types of base conversions, base pair insertions or deletions, and even combinations without the generation of DSBs in a targeted way (37). PEs are composed of Cas9 H840A nickase fused to an engineered reverse transcriptase (RTase) enzyme as well as a prime-editing guide RNA (pegRNA) (**Figure 2**, right panel). The pegRNA contains an sgRNA sequence for Cas9 targeting, a primer binding site (PBS) for RT initiation, and an RT template with the desired edits. To achieve more efficient and precise editing, an additional sgRNA (nicking sgRNA) has been introduced into the PE3 and PE3b systems (37). Although PEs enable precise genome editing with great versatility, the editing efficiency among endogenous sites varies (134, 135). The design of optimal pegRNAs plays a critical role in PE with high efficiency, and numerous efforts have been made to improve the editing efficiency of PE by modifying pegRNAs. For example, ePE was developed by introducing multiple modifications to pegRNAs, which resulted in a marked increase in editing efficiency (136). Nelson et al. reported pegRNAs with enhanced stability and improved editing efficiency, and they did not show increased off-target editing activity (137). xrPE presents substantially enhanced editing efficiency based on appending an RNA motif to prevent pegRNA degradation (138). Li et al. developed spegRNA and apegRNA to increase the PE base-editing and indel editing efficiency by introducing same-sense mutations into the RT template of pegRNA and altering the pegRNA secondary structure, respectively (139). Modification of pegRNAs with G-quadruplexes also effectively improved the editing efficiency of Pes (140). PE4 and PE5 were developed by transient co-expression of an engineered DNA mismatch repair inhibiting protein MLH1 with PE2 and PE3, respectively (141), and further optimization resulted in PEmax with enhanced PE performance. Employing paired pegRNAs that target opposite DNA strands, PRIME-Del has been reported to enable precise genomic deletions of up to 10 kb, and extended expression of PE components can substantially enhance efficiency without compromising precision (142). Zhuang et al. reported an approach named HOPE in which paired pegRNAs encoding the same edits in both sense and antisense DNA strands were used to achieve high editing efficiency as well as high editing purity (143). Using a PE protein and two pegRNAs, twinPE enables efficient gene replacement or excision, large DNA plasmid (>5,000 bp) integration, and targeted sequence inversions (144). A similar strategy has been implemented to enable the donor-free insertion of large DNA sequences by GRAND editing (145). PE-Cas9-based deletion and repair (PEDAR) has been reported to enable precise genomic deletion and replacement of genomic fragments (146). Replacing SpCas9 H840A nickase with PAM-flexible Cas9 variants has been reported to expand the editing modality of PE (147, 148). By generating an all-in-one PE system and a Cas9 nuclease instead of a nickase, Adikusuma et al. showed improved editing efficiency in cultured cells and mouse zygotes (149). Furthermore, for efficient and simple manipulation, researchers have developed computational tools to design pegRNAs or predict pegRNA efficiency (150–152). As a versatile genome editing tool, PEs have been used in cultured mammalian cells (37), plants (135), and model animals (134, 153, 154) to enable the introduction of any small genetic mutation. The off-target effect of PE is relatively low at the same target sites compared with that of Cas9, which may be due to the two additional nucleic acid hybridization steps (nicked target strand–PBS hybridization and 3′ flap–target strand hybridization) that are required for PE (96). Using genomic and transcriptomic sequencing analyses, researchers demonstrated that PE3 did not induce any pegRNA-independent off-target mutations in mammalian cells or zygotes (155–157).

## 3 CRISPR/Cas based gene therapy for IEIs

### 3.1 Progress in CRISPR/Cas gene therapy for genetic diseases

CRISPR/Cas systems enable targeted genome manipulation in a programmable and efficient manner and have remarkable potential for correcting mutations underlying human genetic diseases. Recent progress in the development of advanced gene editing approaches with high activity and precision has paved the way for theories to enter practice.

CRISPR/Cas-mediated gene disruption may be useful for removing disease-causing replicated fragments or GOF mutants. Huntington’s disease (HD) is caused by a dominantly inherited CAG repeat expansion in exon 1 of the huntingtin gene (HTT), which can be removed through CRISPR-Cas9-mediated fragment deletion in patient-derived fibroblasts and mouse models (158, 159). Mutations in the hemoglobin subunit beta gene (HBB), which encodes β-globin, is related to hereditary anemias such as sickle cell disease (SCD) and β-thalassemia. Re-expression of the paralogous γ-globin genes (*HBG1/2*) could ameliorate the severe β-globin disorders. Diminishing the expression of the γ-globin transcriptional repressor or attenuating γ-globin-to-β-globin switching through CRISPR/Cas9-mediated gene disruption resulted in increased γ-globin expression, which ameliorated the pathogenic phenotype (160–166). Clinical trials for the treatment of patients with β-thalassemia and severe sickle cell disease by transfusion of CRISPR/Cas-edited HSCs have been initiated and have displayed exciting results (167, 168). Leber congenital amaurosis 10 (LCA10) is a severe rare genetic eye disease caused by mutations in the CEP290 gene, and SaCas9 was delivered in conjunction with dual gRNAs into a human CEP290 IVS26 knock-in mouse model by a single adeno-associated virus (AAV) to remove the aberrant splice donor generated by the IVS26 mutation of the CEP290 gene (169). The genome editing therapeutic EDIT-101 was initiated by Allergan and Editas Medicine for the treatment of LCA10. By targeting misfolded transthyretin (TTR), CRISPR/Cas9 has been used in the treatment of transthyretin (ATTR) amyloidosis by targeting misfolded transthyretin (TTR), and the safety and pharmacodynamic effects of NTLA-2001 have been evaluated in an ongoing clinical study (170). Splicing mutations responsible for cystic fibrosis (CF) have been successfully repaired using CRISPR/Cas-mediated sequence cleavage (171). CRISPR/Cas gene disruption has also been applied in the treatment of inherited liver diseases. Hereditary tyrosinemia type I (HTI) is a severe inherited metabolic disorder caused by loss-of-function mutation of FAH. Knocking out hydroxyphenylpyruvate dioxygenase (HPD, an upstream enzyme of FAH) has been demonstrated to prevent toxic metabolite accumulation and has been used to treat HTI metabolic disease in Fah^−/−^mice (172). Disruption of GOF mutations in alpha-1 antitrypsin (AAT) by CRISPR/Cas could ameliorate the pathologic liver phenotype (173). Disruption of proprotein convertase subtilisin/kexin type 9 (PCSK9) by delivering CRISPR/Cas into the mouse liver resulted in significant reductions in serum Pcsk9 and total cholesterol levels has been observed (43, 174, 175). CRISPR/Cas-mediated frameshifts have also been exploited to treat DMD, a progressive muscular dystrophy caused by mutations in the dystrophin gene. Dystrophin expression was rescued and the pathogenic phenotype was partially recovered in DMD animals after local or systemic delivery of CRISPR/Cas components (176–179). Transmembrane channel-like 1 (Tmc1) mutation causes hearing loss, and cationic lipid-mediated *in vivo* delivery of Cas9:gRNA RNP complex in the humanized transmembrane channel-like 1 (Tmc1) Beethoven (Bth) mouse model ameliorated the pathogenic phenotype (64).

Replacement of a mutated sequence with the correct fragment *via* the HDR pathway is an attractive strategy for gene therapy. HDR-mediated gene replacement has been used to correct disease-causing point mutations in the β-globin (HBB) gene in HSCs and patient-derived HSPCs to treat β-thalassemia and sickle cell disease (SCD) (63). Martyn et al. reported that the precise insertion of a point mutation *via* CRISPR/Cas-mediated HDR creates a *de novo* binding site for the transcriptional activator GATA1, which drives γ-globin expression and HbF production, thus providing a therapeutic strategy for β-thalassemia (180). Successful modeling and correction of human familial hypercholesterolemia, which causes point mutations, through the CRISPR/Cas9-mediated HDR pathway have been achieved (181). Using ssODNs as a homology donor, gene correction was achieved in Cockayne syndrome (CS) or amyotrophic lateral sclerosis (ALS) patient-derived induced pluripotent stem cells (iPSCs) *via* the CRISPR/Cas9-mediated HDR pathway (182, 183). Furthermore, HDR-mediated mutant gene correction has also been reported in inherited liver diseases, such as HTI and AATD (184–186). CRISPR/Cas HDR-mediated corrections of pathogenic mutations in pre-implantation human embryos have also been reported (187). However, the application of HDR-mediated gene therapy is limited because this method is confined to dividing cells and has a low editing rate and co-delivering donor DNA with CRISPR/Cas components is difficult (188).

Compared with HDR, BE-mediated editing can be applied to both dividing cells and terminally differentiated cell types in a predictable manner. Researchers have delivered ABEs mRNA and sgRNAs targeting Pcsk9 *in vivo* using lipid nanoparticles (LNPs) to introduce PCSK9 loss-of-function mutations in living cynomolgus monkeys, which resulted in significant reductions in the blood levels of PCSK9 and LDL cholesterol (118, 189). Zeng et al. performed base editing using purified A3A (N57Q)-BE3 protein with chemically modified synthetic sgRNAs as RNP complexes targeting the BCL11A erythroid enhancer for fetal hemoglobin (HbF) induction in human CD34 HSPCs (190). By utilizing ABE8e-NRCH, Newby et al. successfully converted an SCD-causing mutation into a nonpathogenic variant in HSPCs derived from patients or humanized SCD mice (120). Villiger et al. delivered SaKKH-BE3 through intravenous injection of dual-AAV plasmids into a PKU mouse model to restore phenylalanine hydroxylase (PAH) enzyme activity and revert the light fur phenotype in these animals (117). Viral delivery of BE3 *in utero* resulted in long-term postnatal persistence of edited cells and reduced plasma PCSK9 and cholesterol levels (191). Song et al. delivered ABE into an adult mouse model of HTI in a non-viral manner to correct a FAH point mutation (192). Using a dual-AAV system, ABE was delivered to muscle cells in a DMD mouse model to correct a nonsense mutation in the dystrophin gene (116). Splice donor sites of the dystrophin gene have been targeted using ABE in human iPSCs and transgenic mice, and this process restores dystrophin expression (193). Efficient and precise correction of pathogenic point mutations involving Marfan syndrome, β-thalassemia, and lamellar ichthyosis has also been performed in human embryos, thus demonstrating the feasibility of curing genetic diseases in human embryos by BEs (104, 119, 194). Delivery of ABEs using a lentivirus to patient-derived fibroblasts or administering AAV to a transgenic mice resulted in the substantial direct correction of the pathogenic mutation underlying Hutchinson-Gilford progeria syndrome (HGPS or progeria) (23). Delivery of BEs into the mouse brain using dual AAVs successfully corrected a mutation that causes Niemann-Pick disease type C, which slows down neurodegeneration and increases lifespan (195). CRISPR DNA BEs were designed to mediate irreversible base substitutions without generating DSBs or requiring donor DNA; thus, in theory, they could enable the manipulation of genomes in a safer and more precise manner. However, BEs can only mediate limited types of editing, while most known disease-associated genetic variants are specific transversions, deletions, insertions, and others (37). Thus, genome engineering tools that can install more types of genetic changes are aspired.

In theory, PEs can correct up to 89% of pathogenic human genetic variants reported in ClinVar (196). Schwank’s al. delivered PE into a mouse model of phenylketonuria (PKU) to repair the disease-causing Pahenu2 mutation *via* the human adenoviral vector 5 (AdV) (197). Utilizing a dual-AAV delivery system, Liu et al. delivered a split-intein PE to enable the correction of AATD pathogenic mutations in mouse liver (198). Zhi et al. developed a dual-AAV delivery strategy that successfully delivered PEs into adult mouse retina for Dnmt1 editing *in vivo* (199). Jang et al. reported that PEs delivered by hydrodynamic injection and AAV enable precise correction of disease-causing mutations and amelioration of disease phenotypes in mouse models of HT1 and LCA without detectable off-target edits (200). By introducing insertions, a PE-mediated reframing strategy has been exploited to restore dystrophin expression in human cardiomyocytes (193). PE-mediated correction of pathogenic point mutations could also be performed in chemically derived hepatic progenitors (CdHs) through a non-viral delivery method to treat genetic liver disease (201). Schene et al. demonstrated that PEs could functionally recover disease-causing mutations in intestinal organoids from patients with DGAT1-deficiency and liver organoids from a patient with Wilson disease (ATP7B) (202). Using optimized PEs, Jiang et al. removed a 1.38-kb pathogenic insertion within the Fah gene and precisely repaired the deletion junction to restore FAH expression in the liver of a tyrosinemia mouse model (146). PEs have also been utilized to correct COL7A1 mutations in recessive dystrophic epidermolysis bullosa (RDEB) patient-derived fibroblasts, and functional rescue was observed (203). Correction of a disease-related mutation in α 1-antitrypsin (A1AT)-deficient patient-derived induced pluripotent stem cells (iPSCs) has been achieved, and guide RNA-independent off-target mutations have not been detected in the genome (155). Due to the large size of PEs, delivery efficiency as well as editing efficiency may represent limitations that significantly impact the therapeutic application of this technology.

### 3.2 CRISPR/Cas gene therapy of IEIs

As most IEIs are caused by loss-of-function mutations, a viral-mediated gene addition strategy has been developed for T-cell therapies. A retroviral strategy was the first class used, and although long-term immune reconstitution was achieved in patients subjected to retroviral gene transfer, insertional mutagenesis and oncogene transactivation associated with the use of retroviral vectors were observed in gene therapy trials for X-SCID, CGD, and WAS (204–207). To circumvent these adverse events, an improved generation of gene delivery vectors, referred to as SIN vectors, has been developed. Compared with gammaretroviral vectors, which integrate primarily within transcriptional regulatory elements, lentiviral vectors preferentially integrate into active transcription units and, hence, have a better safety profile. Furthermore, lentiviral vectors can be produced at much higher titers and may enable effective transduction in a wide range of cell types; thus, lentiviral vectors have become the most commonly used vectors for the insertion of therapeutic genes into HSPCs in both research and clinical trials (18, 28, 29). Lentiviral vector-mediated gene transfer has been successfully applied in gene therapy for some IEIs, including X-SCID, ADA-SCID, X-CGD, and WAS; moreover, effective immune reconstitution has been observed and no adverse events related to the vector have been reported in these clinical trials (208–213). These positive results suggest that lentiviral-based gene addition therapy provides sustained clinical benefits for patients with IEIs. However, for genes involved in some IEIs caused by dominant-negative mutations or those that require precise regulation (e.g., forkhead box P3, FOXP3; Bruton tyrosine kinase, BTK; CD40 ligand, CD40LG), gene augmentation therapy is not appropriate; however, *in situ* correction of the endogenous defective gene would allow for physiological regulation of gene transcription and protein expression.

As a promising therapeutic approach for treating monogenic hematological diseases, such as IEIs, CRISPR/Cas-mediated gene disruption has been employed to generate animal models while CRISPR/Cas-mediated gene replacement through HDR enables precise correction of disease-causing genes. Targeted integration of one correct copy of a gene of interest in its physiological transcriptional regulation context is particularly important for the treatment of disorders caused by mutations in tightly regulated genes. The CRISPR/Cas-based gene therapy strategy has been applied to the modeling and correction of many forms of IEIs, including SCID, immune dysregulation, polyendocrinopathy, enteropathy, X-linked (IPEX), X-linked MAGT1 deficiency with increased susceptibility to EBV-infection and N-linked glycosylation defect (XMEN), X-CGD, and WAS. **Table 1** listed known IEIs with CRISPR/Cas technology has been applied in their studies.

**Table 1:** List of the known IEIs with CRISPR-Cas technology has been applied in their studies.

IEIs	Genes	Editing Strategy	Target Cells	Delivery	Refs
XHIGM	CD40LG	CRISPR/Cas9 mediated integration of a normal copy of the CD40L cDNA	HSCs, patient-derived T cells	AAV6	(234)
XHIGM	CD40LG	CRISPR/Cas9 mediated insertion of a 5’‐truncated *CD40LG* cDNA within the first intron of the endogenous gene	HSCs, patient-derived T cells	AAV6	(235)
XLA	BTK	CRISPR/Cas9 mediated integration of a codon optimized BTK cDNA into its endogenous locus	HSPCs, Jurkat, K562, Ramos cells,	Electroporation, AAV6	(236)
XLP	*SH2D1A*	CRISPR/Cas9 or CRISPR/Cas12a drive insertion of SAP cDNA at the first exon of the SH2D1A locus	PrimaryT cells	Nucleofection, AAV6	(237)
SCID	JAK3	CRISPR/Cas9 nuclease or CRISPR/Cas9 D10A nickase mediated HDR	iPSCs	Nucleofection	(214)
SCID	RAG2	CRISPR/Cas9 mediated multiplex HDR	HSPCs	AAV6	(215)
X-SCID	IL2RG	CRISPR/Cas9 mediated integration of a cDNA into the endogenous locus	LT-HSCs	AAV6	(216)
X-SCID	IL2RG	CRISPR/Cas9 mediated knock-in of a corrective IL2RG cDNA into the affected locus	HSPC	AAV6	(217)
X-SCID	IL2RG	CRISPR/Cas9 mediated disruption of IL2Rg	Rabbit embryos	Microinjection of gRNA and Cas9 mRNA	(218)
SCID	IL2RG, RAG2	CRISPR/Cas9 mediated gene knockout	Rat zygotes	Electroporation of gRNA and Cas9 mRNA	(219)
SCID	IL2RG, RAG2	CRISPR/Cas9 mediated double knockout	Mice zygotes	Microinjection of gRNA and Cas9 mRNA	(220)
SCID	IL2RG	CRISPR/Cas9 mediated gene knockout	pig embryos	Microinjection of gRNA and Cas9 mRNA, nucleofection	(221–223)
SCID	IL2RG	CRISPR/Cas9 mediated gene knockout	Hamster embryos	pronuclear injection of gRNA and Cas9 mRNA	(224)
IPEX	FOXP3	CRISPR/Cas9 mediated integration of FOXP3 cDNA into the endogenous locus	Patient derived HSPCs	AAV6	(225)
XMEN	MAGT1	CRISPR/Cas9 mediated HDR to insert a correct MAGT1 cDNA into the endogenous locus	HSPCs	AAV6	(226)
X-CGD	CYBB	CRISPR/Cas9 mediated HDR for gene repair using ssODN	Patient derived HSPCs	Electroporation	(227)
X-CGD	CYBB	CRISPR/Cas9 mediated insertion of CYBB cDNA at endogenous locus	Patient derived HSPCs	AAV6	(228)
X-CGD	CYBB	CRISPR/Cas9 mediated gene replacement and insertion	Patient-specific iPSCs	nucleofection	(229)
X-CGD	CYBB	CRISPR/Cas9 mediated gene knockout	Mouse zygotes	microinjection of gRNA and Cas9 mRNA	(230)
WAS	WAS	CRISPR/Cas9 mediated HDR for integrating a full-length cDNA into endogenous locus	Patient derived HSPCs, LT-HSCs	AAV6, electroporation	(231)
WAS	WAS	CRISPR/Cas9 mediated gene knockout	Rabbit embryos	microinjection of Cas9 mRNA and a pair of sgRNAs	(232)
WAS	WAS	CRISPR/Cas9 mediated gene knockout	primary T cells	Vapor nanobubble (VNB) photoporation	(233)

XHIGM, X-linked hyper-IgM syndrome; XLA, X-linked agammaglobulinemia; XLP, X-Linked Lymphoproliferative Disease; SCID, Severe combined immunodeficiency; X- SCID, X-linked severe combined immunodeficiency; IPEX, Immune dysregulation, polyendocrinopathy, enteropathy, X-linked syndrome; XMEN, X-linked immunodeficiency with magnesium defect, EBV infection, and neoplasia; X-CGD, X-linked chronic granulomatous disease; WAS: Wiskott-Aldrich syndrome; HSCs, Hematopoietic stem cell; HSPCs, hematopoietic stem and progenitor cells; LT-HSCs, long-term hematopoietic stem cells; iPSCs, induced pluripotent stem cells; MSCs, mesenchymal stem cells.

SCID is the most profound IEI affecting cellular and humoral immunity, and comprises a group of disorders caused by mutations in genes involved in lymphocyte development. By employing Cas9-mediated HDR strategy, Chang et al. achieved correction of JAK3 mutations in SCID patient-specific induced pluripotent stem cells (iPSCs) and observed restoration of T cell development (214). Recently, Iancu et al. reported a CRISPR-mediated multiplex HDR strategy for disease modeling and correction in SCID patient-derived HSPCs; biallelic knockout of genes was utilized to generate a disease model while knockin/knockout strategy was used for single-allelic gene correction (215). The most common form of SCID, X-SCID, is caused by mutations in the genes encoding interleukin 2 receptor gamma (IL2RG). Integration of IL2RG cDNA into the endogenous start site in human long-term hematopoietic stem cells (LT-HSCs) using the CRISPR/Cas9-mediated HDR strategy has led to the functional correction of disease-causing mutations throughout the gene (216). In a preclinical study, functional integration of IL2RG was achieved by integrase-defective lentiviral vector (IDLVs)-mediated delivery of a donor DNA template and sgRNA in SCID-X1–Cas9^+/−^ HSPCs with low cytotoxicity (217). In addition to treatment, CRISPR/Cas technology has been used to generate SCID animal models, including mice, rabbits, rats, pigs, and hamsters, for basic and translational research (218–224).

IPEX syndrome is a monogenic immune disease caused by mutations in FOXP3 that selectively affect the function of regulatory T cells (Tregs). By combining the CRISPR/Cas9 system with a DNA repair homology donor, Goodwin et al. enabled efficient and precise insertion of the FOXP3 cDNA into the endogenous locus of patient derived HSPCs, and the normal differentiation potential of edited HSPCs was maintained both *in vitro* and *in vivo* in immunodeficient mice (225).

XMEN is a recently described IEI caused by an X-linked MAGT1 deficiency, and it is marked by defective T cells and natural killer (NK) cells. Brault et al. reported an *ex vivo* targeted gene therapy approach using an optimized CRISPR/Cas HDR strategy to insert a therapeutic MAGT1 gene into the endogenous locus, and it presented high genetic correction efficiency and functional rescue in engrafted mice (226).

X-CGD is an immunodeficiency caused by mutations in the CYBB gene, which encodes the gp91^phox^ protein. CRISPR/Cas9-mediated HDR was employed for CYBB gene correction in HSPCs and functional restoration was achieved (227–229). Sweeney et al. reported that targeted insertion of CYBB exon 2-13 by CRISPR/Cas HDR at the endogenous CYBB exon 2 site in patient HSPCs fully restored gp91^phox^ expression and transient inhibition of the NHEJ pathway increased the gene correction rate (228). De Ravin et al. repaired a frequent mutation of the CYBB gene in HSPCs from X-CGD patients through CRISPR/Cas9-mediated HDR with an ssODN donor and achieved functional restoration up to 5 months after transplantation of the corrected HSPCs into NSG immunodeficient mice (227). Gene replacement of CYBB exon 5 mutations using CRISPR/Cas HDR has also been administered administrated in patient-derived iPSCs (229). To enable *in vivo* assessments of gene and cell therapy strategies for treating CGD, a mouse model of X-CGD was established using the CRISPR/Cas system by targeting CYBB exon 1 or exon 3 (230).

Wiskott–Aldrich syndrome (WAS) is an X-linked recessive immunodeficiency disease caused by mutations in the WAS gene encoding WAS protein (WASp). Rai et el. inserted a therapeutic WAS cDNA in-frame with its endogenous translation start codon in patient-derived HSPCs *via* CRISPR/Cas9-mediated HDR, and full rescue of WASp expression and correction of functional defects were observed (231). Microinjection of Cas9 mRNA and a pair of sgRNAs targeting exons 2 and 7 of WAS into rabbit embryos was performed to generate a model exhibiting symptoms similar to those of WAS patients for preclinical studies (232). In a proof-of-concept study, the WAS gene was disrupted in human primary T cells using the CRISPR/Cas9 system through an advanced delivery method (233).

X-linked hyper-immunoglobulin M (hyper-IgM) syndrome (XHIGM) is an IEI caused by mutations in CD40 ligand (CD40L) that affect immunoglobulin class-switch recombination and somatic hypermutation. Utilizing either the TALEN or CRISPR/Cas9 platforms, Kuo et al. integrated a normal copy of the CD40L cDNA with the endogenous CD40L promoter to maintain the physiological expression of CD40L in primary human HSCs and XHIM-patient-derived T cells (234). In a preclinical study, Vavassori et al. developed a universal CRISPR/Cas editing strategy for effective integration of a corrective CD40L cDNA within its endogenous locus and achieved effective T cell correction and selection by optimizing the editing strategy. The therapeutic potential of T-cell and HSPC therapies was further investigated in the HIGM1 mouse model (235).

X-linked agammaglobulinemia (XLA) is a monogenic IEI caused by a loss-of-function mutation in Bruton’s tyrosine kinase (BTK) gene, and it is characterized by reduced mature B lymphocytes. Gray and colleagues reported that precise integration of a corrective, codon-optimized cDNA along with a truncated terminal intron of the BTK gene into its endogenous locus by CRISPR/Cas-mediated HDR substantially elevated BTK expression to potentially therapeutic levels in cell lines and human HSPCs (236).

X-Linked Lymphoproliferative Disease (XLP) is a rare IEI that is caused by a mutation in the signaling lymphocyte activation molecule (SLAM)-associated protein (SAP) protein-coding gene *SH2D1A*. Targeted insertion of SAP cDNA at the first exon of the SH2D1A locus was efficiently achieved using TALEN, CRISPR/Cas9, or CRISPR/Cas12a, and SAP protein expression and SAP-dependent immune function were restored in patient-derived T cells (237).

**Figure 3:**
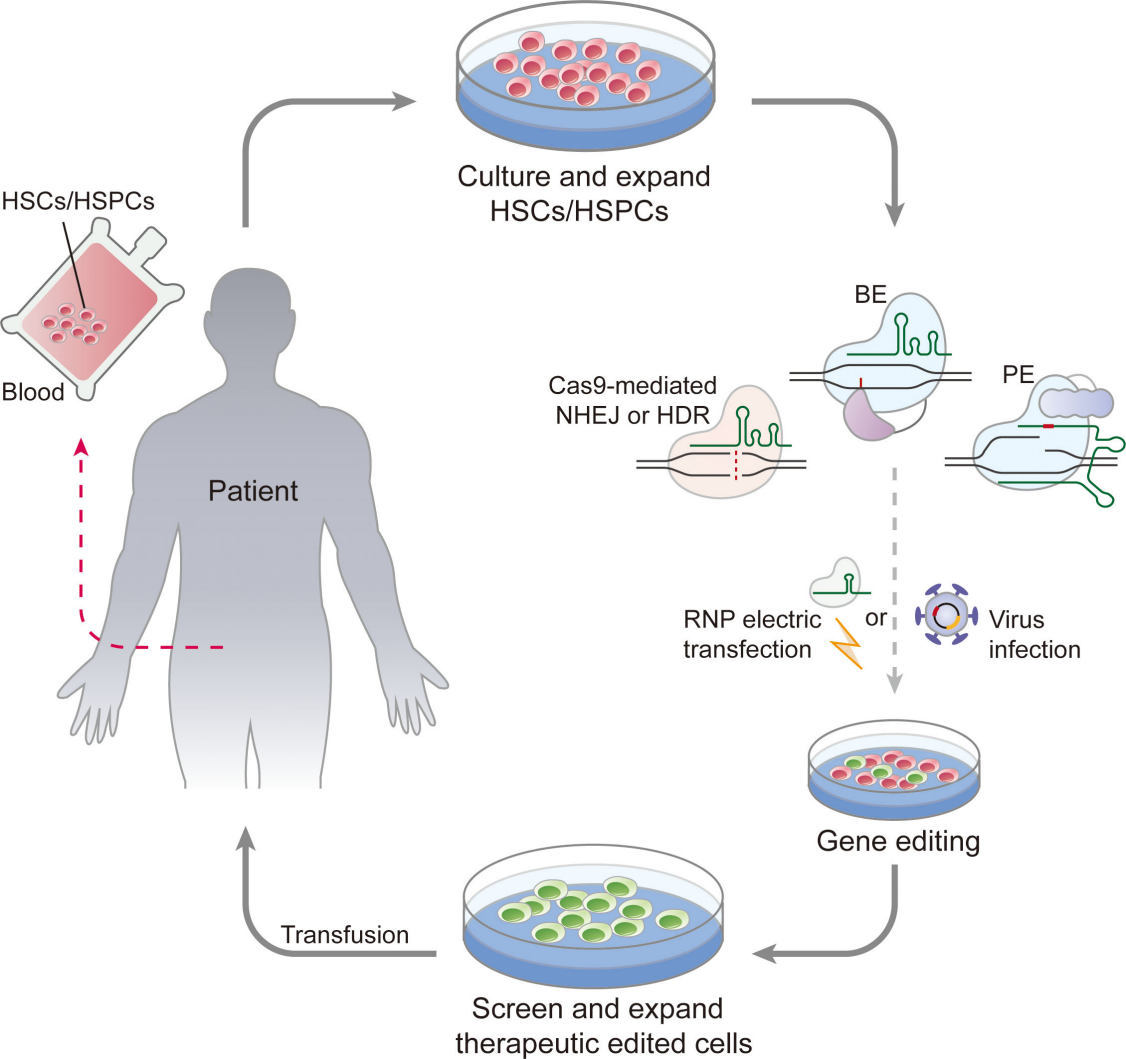
Work flow of CRISPR/Cas gene editing in gene therapy of IEIs. Autologous HSCs collected from patients undergo *ex vivo* culture and CRISPR/Cas editing, after screening and expansion, therapeutic edited cells are transfusion into conditioned patients for immune system reconstruction. CRISPR/Cas gene editing agents could be delivered into HSCs in forms of RNP, “all RNA”, or AAV vector to enable efficient pathogenic gene correction.

Compared with the inefficient HDR-mediated site-specific integration of target genes, NHEJ-based homology-independent targeted integration (HITI) with improved integration frequencies has been utilized to achieve robust site-specific transgene integration at a clinically relevant genetic locus in human mobilized peripheral blood HSPCs (238). This strategy provides an alternative to HDR-based gene integration in human HSPCs. Together, these studies demonstrated that CRISPR/Cas gene editing in HSCs is highly promising for correcting disease-causing mutations in IEIs. (**Figure 3**)

BEs enable single-base substitutions without producing DSBs, while PEs enable precise base conversions, insertions, deletions, and even combinations without inducing DSBs, and both of these editors are promising in genetic disease modeling and correction. Compared with BEs, PEs induce more accurate and precise on-target editing, and more importantly, PEs enable multiple types of editing. Gene therapy studies based on BEs or PEs for IEI treatment have not been reported yet. Thus, to identify potential targets for BE- and/or PE-mediated gene correction, disease-causing genes need to be carefully sequenced and sgRNAs and pegRNAs should be designed appropriately to enable efficient editing.

### 3.3 Delivery of CRISPR/Cas gene editing reagents into HSCs

The targeted and efficient delivery of editing reagents plays an important role in gene therapy. Mainstream methods for delivering editing components into HSCs are based on electroporation, viral vectors, or nanoparticles (239). Electroporation is an effective method for delivering editing reagents into a large number of HSCs; however, voltage-induced cell toxicity should be considered (240). Lentiviruses and AAVs are mainly used to deliver editing elements into HSCs, with lentiviruses integrating DNA fragments into genome and AAV presenting a limited cargo capacity for CRISPR/Cas system packaging (239). Developing non-integrating viral vectors and compact CRISPR/Cas systems could provide alternatives to overcome these challenges. Nanoparticles are a class of materials that can encapsulate therapeutic nucleic acids for targeted delivery, and lipid nanoparticles (LNPs) are the most common and have been investigated for their ability to deliver CRISPR/Cas systems for therapeutic purposes (241). A protein-based nanoparticle system (Selective Endogenous eNcapsidation for cellular Delivery, SEND) has been developed to enable efficient delivery of CRISPR/Cas editing reagents into mammalian cells (242). The recently developed delivery strategy named SORT (selective ORgan targeting (SORT) allows lipid nanoparticles to encapsulate gene therapy reagents to be accurately delivered in an organ-specific manner (243). Compared with electroporation, delivery strategies based on viral vectors and nanoparticles can be utilized in both *ex vivo* and *in vivo* gene therapy.

CRISPR/Cas components are usually delivered into HSCs in two major forms: the RNP complex of purified Cas protein precomplexed with sgRNAs and “all RNA” delivery with Cas mRNA with or without chemically modified sgRNAs. Hendel et al. reported that chemically modified sgRNAs could enhance genome editing in both human primary T cells and HSPCs; use of RNA- or RNP-based delivery method further improved editing (244). Protection of both sgRNA termini with chemically modified nucleotides increases the stability of sgRNA and renders the ‘all RNA’-based CRISPR/Cas9 system highly effective in primary HSPCs and T cells (245). By optimizing and comparing several delivery strategies, Lattanzi et al. demonstrated that the RNP-based delivery of CRISPR/Cas editing reagents exhibited a good balance between cytotoxicity and genomic rearrangement efficiency (246). The donor template for HDR-mediated gene correction can be delivered to HSCs with AAV6-based vectors or ssODNs. Co-delivery of an AAV6 donor in combination with CRISPR/Cas RNP enabled the precise correction of WAS mutations in up to 60% of human HSPCs (231). A recent study reported that FOXP3 cDNA was delivered by AAV6 with the high-fidelity Cas9 variant HiFi-Cas9:sgRNA RNP into HSPCs to correct pathogenic mutations with high specificity (225). AAV6 donor templates can mediate transgene integration at up to ~4 kb in size, thus encompassing an expression cassette for a reporter gene, which is important for the enrichment of HSCs with precisely targeted integration (245). In comparison to AAV6, ssODNs are usually ~50–200 bp in total size and can be useful donor templates for single point mutation correction. Phosphorothioate-modified ssODNs of the CYBB gene fragment have been delivered with the CRISPR/Cas RNP complex to patient-derived HSPCs to repair a mutation causing X-CGD. Transplanting gene-repaired X-CGD HSPCs into NSG mice resulted in efficient engraftment and production of functional mature human myeloid and lymphoid cells for up to five months (227).

### 3.4 Safety concerns of CRISPR/Cas gene therapy of IEIs

In terms of safety, CRISPR/Cas gene therapy avoids the need for semi-random integration of viral vectors, thereby minimizing the risk of integration-activated oncogenesis. One of the major concerns associated with CRISPR/Cas gene therapy is the potential off-target effects; many efforts have been made for off-target prediction, characterization, and reduction in both cell lines and human HSPCs (247, 248). Off-target prediction by in silico predictive algorithms is powerful and useful for potential genome-wide off-target prediction, and cell-based and cell-free detection methods have been developed to determine the distribution and frequency of off-target events. Protein engineering, gRNA modification, and delivery methods have been exploited to limit the off-target effect (247). CRISPR/Cas systems introduce DSBs in the DNA and thus induce HDR, and indels induced by DSBs at the target site can be minimized by inhibiting the NHEJ pathway to enhance HDR (72, 73). BEs and PEs are DSB-independent editing strategies, and Cas-independent genomic and transcriptomic off-targets of BEs have been reported (104–106). Rational nucleotide deaminase engineering could circumvent this and provide BEs with reduced off-target editing activity, while maintaining robust on-target editing efficiency (77, 89). Additionally, delivering BEs, such as RNPs, could also greatly decrease off-target editing while maintaining comparable on-target editing (115). PEs were demonstrated to have high specificity since neither off-target DNA nor off-target RNA was detected in mammalian cells or zygotes (155–157).

### 3.5 CRISPR/Cas based clinical trails

CRISPR/Cas gene therapy has been considered a promising strategy to treat many incurable genetic and nongenetic disorders, including hereditary blood diseases, ocular diseases, neuromuscular disorders, metabolic disorders, cancers, and acquired immunodeficiency syndrome. Currently initiated clinical trials based on CRISPR/Cas technology are both *ex vivo* and *in vivo*. *Ex vivo* gene therapy infuses cultured cells into patients after editing by CRISPR/Cas, and *in vivo* gene therapy delivers CRISPR/Cas gene editing reagents into patients to facilitate gene modification at pathogenic sequences. The current active CRISPR/Cas-based clinical trials are listed in **Table 2**.

**Table 2:** Currently active clinical trials based on CRISPR/Cas technology.

NCT Number	Title	Status	Conditions	Interventions	Phase	Enrollment	Sponsor/Collaborators
NCT04774536	Transplantation of Clustered Regularly Interspaced Short Palindromic Repeats Modified Hematopoietic Progenitor Stem Cells (CRISPR_SCD001) in Patients With Severe Sickle Cell Disease	Not yet Recruiting	Sickle Cell Disease	CRISPR_SCD001	I/II	9	Mark Walters, MD; University of California, Los Angeles; University of California, Berkeley; University of California, San Francisco
NCT03057912	A Safety and Efficacy Study of TALEN and CRISPR/Cas9 in the Treatment of HPV-related Cervical Intraepithelial Neoplasia#	Unknown Status	Human Papillomavirus- Related Malignant Neoplasm	CRISPR/Cas9	I	60	First Affiliated Hospital, Sun Yat- Sen University; Jingchu University of Technology
NCT04426669	A Study of Metastatic Gastrointestinal Cancers Treated With Tumor Infiltrating Lymphocytes in Which the Gene Encoding the Intracellular Immune Checkpoint CISH Is Inhibited Using CRISPR Genetic Engineering	Recruiting	Gastrointestinal Epithelial Cancer; Gastrointestinal Neoplasms; Cancer of Gastrointestinal Tract; Gastrointestinal Cancer; Colo-rectal Cancer; Pancreatic Cancer; Gall Bladder Cancer; Colon Cancer; Esophageal Cancer; Stomach Cancer	Tumor-Infiltrating Lymphocytes (TIL)	I/II	20	Intima Bioscience, Inc.; Masonic Cancer Center, University of Minnesota
NCT03164135	Safety of Transplantation of CRISPR CCR5 Modified CD34+ Cells in HIVinfected Subjects With Hematological Malignances	Unknown Status	HIV-1-infection	CCR5 gene modification	N/A	5	Affiliated Hospital to Academy of Military Medical Sciences; Peking University; Capital Medical University
NCT04560790	Safety and Efficacy of CRISPR/Cas9 mRNA Instantaneous Gene Editing Therapy to Treat Refractory Viral Keratitis	Completed	Viral Keratitis; Blindness Eye; Herpes Simplex Virus Infection; Cornea	BD111 Adult single group Dose	N/A	3	Shanghai BDgene Co., Ltd.; Eye & ENT Hospital of Fudan University
NCT04990557	CRISPR/Cas9-modified Human T Cell ( PD-1and ACE2 Knockout Engineered T Cells ) for Inducing Longterm Immunity in COVID-19 Patients	Not yet Recruiting	COVID-19 Respiratory Infection	PD-1 and ACE2 Knockout T Cells	I/II	16	Mahmoud Ramadan mohamed Elkazzaz; Kafrelsheikh University
NCT03545815	Study of CRISPR-Cas9 Mediated PD-1 and TCR Gene-knocked Out Mesothelin-directed CAR-T Cells in Patients With Mesothelin Positive Multiple Solid Tumors.	Unknown Status	Solid Tumor, Adult	anti-mesothelin CAR-T cells	I	10	Chinese PLA General Hospital
NCT04037566	CRISPR (HPK1) Edited CD19-specific CAR-T Cells (XYF19 CAR-T Cells) for CD19+ Leukemia or Lymphoma.	Recruiting	Leukemia Lymphocytic Acute (ALL) in Relapse; Leukemia Lymphocytic Acute (All) Refractory	XYF19 CAR-T cell	I	40	Xijing Hospital; Xi'An Yufan Biotechnology Co.,Ltd
NCT05566223	Phase 1/2 Study of CISH Inactivated TILs in the Treatment of NSCLC	Not yet Recruiting	Carcinoma, Non-Small-Cell Lung; Metastatic Non Small Cell Lung Cancer; Stage IV Non-small Cell Lung Cancer; Squamous Cell Lung Cancer; Adenocarcinoma of Lung; Large Cell Lung Cancer	CISH Inactivated TIL	I/II	70	Intima Bioscience, Inc.
NCT04244656	A Safety and Efficacy Study Evaluating CTX120 in Subjects With Relapsed or Refractory Multiple Myeloma	Active, not Recruiting	Multiple Myeloma	CTX120	I	26	CRISPR Therapeutics AG; CRISPR Therapeutics
NCT03655678	A Safety and Efficacy Study Evaluating CTX001 in Subjects With Transfusion- Dependent β-Thalassemia	Active, not Recruiting	Beta-Thalassemia	CTX001	II/III	45	Vertex Pharmaceuticals Incorporated; CRISPR Therapeutics
NCT05477563	Evaluation of Efficacy and Safety of a Single Dose of CTX001 in Participants With Transfusion-Dependent #- Thalassemia and Severe Sickle Cell Disease	Recruiting	Beta-Thalassemia; Thalassemia; Sickle Cell Disease	CTX001	III	12	Vertex Pharmaceuticals Incorporated; CRISPR Therapeutics
NCT04438083	A Safety and Efficacy Study Evaluating CTX130 in Subjects With Relapsed or Refractory Renal Cell Carcinoma (COBALT-RCC)	Recruiting	Renal Cell Carcinoma	CTX130	I	107	CRISPR Therapeutics AG; CRISPR Therapeutics
NCT04502446	A Safety and Efficacy Study Evaluating CTX130 in Subjects With Relapsed or Refractory T or B Cell Malignancies (COBALTLYM)	Recruiting	T Cell Lymphoma	CTX130	I	45	CRISPR Therapeutics AG; CRISPR Therapeutics
NCT04035434	A Safety and Efficacy Study Evaluating CTX110 in Subjects With Relapsed or Refractory B-Cell Malignancies (CARBON)	Recruiting	B-cell Malignancy; Non- Hodgkin Lymphoma; B-cell Lymphoma; Adult B Cell ALL	CTX110	I	143	CRISPR Therapeutics AG; CRISPR Therapeutics
NCT04637763	CRISPR-Edited Allogeneic Anti-CD19 CAR-T Cell Therapy for Relapsed/Refractory B Cell Non- Hodgkin Lymphoma	Recruiting	Lymphoma, Non-Hodgkin; Relapsed Non Hodgkin Lymphoma; Refractory B-Cell Non-Hodgkin Lymphoma;	CB-010	I	50	Caribou Biosciences, Inc.
NCT03745287	A Safety and Efficacy Study Evaluating CTX001 in Subjects With Severe Sickle Cell Disease	Active, not Recruiting	Sickle Cell Disease; Hematological Diseases; Hemoglobinopathies	CTX001	II/III	45	Vertex Pharmaceuticals Incorporated; CRISPR Therapeutics
NCT03728322	iHSCs With the Gene Correction of HBB Intervent Subjests With β-thalassemia Mutations	Unknown Status	Thalassemia	iHSCs treatment	early I	12	Allife Medical Science and Technology Co., Ltd.
NCT04925206	A Safety and Efficacy Study Evaluating ET-01 in Subjects With Transfusion Dependent β-Thalassaemia	Active, not Recruiting	Transfusion Dependent Beta- Thalassaemia	ET-01	I	8	EdiGene (GuangZhou) Inc.
NCT04557436	TT52CAR19 Therapy for Bcell Acute Lymphoblastic Leukaemia (B-ALL)	Recruiting	B Acute Lymphoblastic Leukemia	PBLTT52CAR19	I	10	Great Ormond Street Hospital for Children NHS Foundation Trust; University College, London
NCT03747965	Study of PD-1 Gene-knocked Out Mesothelin-directed CAR-T Cells With the Conditioning of PC in Mesothelin Positive Multiple Solid Tumors	Unknown Status	Solid Tumor, Adult	Mesothelin- directed CAR-T Cells	I	10	Chinese PLA General Hospital
NCT05565248	An Open-Label, FIH Study Evaluating the Safety, Tolerability, and Efficacy of VCTX211 Combination Product in Subjects With T1D	Not yet Recruiting	Diabetes Mellitus, Type 1; Metabolic Disease; Glucose Metabolism Disorders; Endocrine System Diseases; Autoimmune Diseases; Immune System Diseases	VCTX211	I/II	40	CRISPR Therapeutics AG; ViaCyte; CRISPR Therapeutics
NCT03166878	A Study Evaluating UCART019 in Patients With Relapsed or Refractory CD19+ Leukemia and Lymphoma	Unknown Status	B Cell Leukemia; B Cell Lymphoma	UCART019	I/II	80	Chinese PLA General Hospital
NCT05210530	An Open-Label, FIH Study Evaluating the Safety and Tolerability of VCTX210A Combination Product in Subjects With T1D	Recruiting	Diabetes Mellitus, Type 1; Metabolic Disease; Glucose Metabolism Disorders; Endocrine System Diseases; Autoimmune Diseases; Immune System Diseases	VCTX210A unit	I	10	CRISPR Therapeutics AG; ViaCyte; CRISPR Therapeutics
NCT05356195	Evaluation of Safety and Efficacy of CTX001 in Pediatric Participants with Transfusion-Dependent #- Thalassemia (TDT)	Recruiting	Beta-Thalassemia; Thalassemia; Sickle Cell Disease	CTX001	III	12	Vertex Pharmaceuticals Incorporated; CRISPR Therapeutics
NCT05329649	Evaluation of Safety and Efficacy of CTX001 in Pediatric Participants With Severe Sickle Cell Disease (SCD)	Recruiting	Sickle Cell Disease; Hydroxyurea Failure; Hemoglobinopathies; Hematological Diseases	CTX001	III	12	Vertex Pharmaceuticals Incorporated; CRISPR
NCT03081715	PD-1 Knockout Engineered T Cells for Advanced Esophageal Cancer	Completed	Esophageal Cancer	PD-1 Knockout T Cells	N/A	16	Hangzhou Cancer Hospital; Anhui Kedgene Biotechnology Co.,Ltd
NCT05144386	Study of EBT-101 in Aviremic HIV-1 Infected Adults on Stable ART	Recruiting	HIV-1-infection	EBT-101	I	9	Excision BioTherapeutics
NCT03398967	A Feasibility and Safety Study of Universal Dual Specificity CD19 and CD20 or CD22 CAR-T Cell Immunotherapy for Relapsed or Refractory Leukemia and Lymphoma	Unknown Status	B Cell Leukemia; B Cell Lymphoma	Universal Dual Specificity CD19 and CD20 or CD22 CAR-T Cells	I/II	80	Chinese PLA General Hospital
NCT04601051	Study to Evaluate Safety, Tolerability, Pharmacokinetics, and Pharmacodynamics of NTLA-2001 in Patients With Hereditary Transthyretin Amyloidosis With Polyneuropathy (ATTRv- PN) and Patients With Transthyretin Amyloidosis- Related Cardiomyopathy (ATTR-CM)	Recruiting	Transthyretin-Related (ATTR) Familial Amyloid Polyneuropathy; Transthyretin-Related (ATTR) Familial Amyloid Cardiomyopathy; Wild-Type Transthyretin Cardiac Amyloidosis	NTLA-2001	I	74	Intellia Therapeutics
NCT04819841	Gene Correction in Autologous CD34+ Hematopoietic Stem Cells (HbS to HbA) to Treat Severe Sickle Cell Disease	Recruiting	Sickle Cell Disease	GPH101	I/II	15	Graphite Bio, Inc.
NCT05066165	Study Investigating NTLA- 5001 in Subjects with Acute Myeloid Leukemia	Active, not Recruiting	Acute Myeloid Leukemia	NTLA-5001	I/II	54	Intellia Therapeutics
NCT04976218	TGF#R-KO CAR-EGFR T Cells in Previously Treated Advanced EGFR- positive Solid Tumors	Recruiting	Solid Tumor, Adult; EGFR Overexpression	Biological: TGFβR-KO CAREGFR T Cells	I	30	Chinese PLA General Hospital
NCT02793856	PD-1 Knockout Engineered T Cells for Metastatic Nonsmall Cell Lung Cancer	Completed	Metastatic Non-small Cell Lung Cancer	PD-1 Knockout T Cells	I	12	Sichuan University; Chengdu MedGenCell, Co., Ltd.
NCT04767308	Safety and Efficacy of CT125A Cells for Treatment of Relapsed/Refractory CD5+ Hematopoietic Malignancies	Not yet Recruiting	CD5+ Relapsed/ Refractory Hematopoietic Malignancies; Chronic Lymphocytic Leukemia (CLL); Mantle Cell Lymphoma (MCL); Diffuse Large B-cell Lymphoma (DLBCL); Follicular Lymphoma (FL); Peripheral T-cell Lymphomas (PTCL)	CT125A cells	early I	18	Huazhong University of Science and Technology; Shanghai IASO Biotechnology Co., Ltd
NCT04417764	TACE Combined With PD-1 Knockout Engineered T Cell in Advanced Hepatocellular Carcinoma.	Unknown Status	Advanced Hepatocellular Carcinoma	PD-1 knockout engineered T Cells	I	10	Central South University
NCT05397184	Study of Base Edited CAR7 T Cells to Treat T Cell Malignancies (TvT CAR7)	Recruiting	Relapsed/Refractory T-cell Acute Lymphoid Leukaemia	Cryopreserved BE CAR7 T cells (BE752TBCCLCAR7PBL)	I	10	Great Ormond Street Hospital for Children NHS Foundation Trust; UCL Great Ormond Street Institute of Child Health; Medical Research Council
NCT05143307	Long-Term Follow-Up Study of HIV-1 Infected Adults Who Received EBT-101	Enrolling by invitation	HIV-1-infection	EBT-101	I	9	Excision BioTherapeutics
NCT05120830	NTLA-2002 in Adults With Hereditary Angioedema (HAE)	Recruiting	Hereditary Angioedema	NTLA-2002	I/II	55	Intellia Therapeutics
NCT05514249	Treatment of a Single Patient With CRD- TMH-001	Active, not Recruiting	Duchenne Muscular Dystrophy	CRD-TMH-001	I	1	Cure Rare Disease, Inc; University of Massachusetts, Worcester
NCT03044743	PD-1 Knockout EBV-CTLs for Advanced Stage Epstein- Barr Virus (EBV) Associated Malignancies	Unknown Status	Stage IV Gastric Carcinoma; Stage IV Nasopharyngeal Carcinoma; T-Cell Lymphoma Stage IV; T-Cell Lymphoma Stage IV; Stage IV Diffuse Large B- Cell Lymphoma	Fludarabine; Cyclophosphamide; Interleukin-2	I/II	20	Yang Yang; The Affiliated Nanjing Drum Tower Hospital of Nanjing University Medical School
NCT05444894	EDIT-301 for Autologous Hematopoietic Stem Cell Transplant (HSCT) in Participants With Transfusion-Dependent Beta Thalassemia (TDT)	Recruiting	Transfusion Dependent Beta Thalassemia; Hemoglobinopathies; Thalassemia Major; Thalassemia Intermedia	EDIT-301	I/II	6	Editas Medicine, Inc.
NCT03872479	Single Ascending Dose Study in Participants With LCA10	Recruiting	Leber Congenital Amaurosis 10; Inherited Retinal Dystrophies; Eye Diseases, Hereditary; Retinal Disease; Retinal Degeneration; Retinal Degeneration; Eye Disorders Congenital	EDIT-101	I/II	34	Editas Medicine, Inc.

N/A, Not Applicable.

The first in-human CRISPR/Cas clinical trial was performed at Sichuan University’s West China Hospital to treat advanced non-small cell lung cancer by targeting the PD-1 gene using CRISPR/Cas9 (ClinicalTrials.gov NCT02793856). According to the reported results, the safety and feasibility were proven, and they demonstrated that a more advanced gene editing strategy to improve efficacy is required in future trials (249). Several phase I/II clinical trials to treat carcinoma by targeting PD-1 with CRISPR/Cas have been initiated (ClinicalTrials.gov NCT02793856, NCT03747965, NCT04417764, and NCT03545815). CRISPR/Cas has also been used to generate chimeric antigen receptor (CAR) T-cells, and clinical trials have been initiated for the immunotherapy of hematologic malignancies and solid tumors (ClinicalTrials.gov NCT03166878, NCT03747965). CRISPR/Cas-mediated gene disruption can be used to alter virus host receptor expression, and clinical trials for antiviral therapeutics against infectious diseases AIDs and COVID-19 have also been registered by targeting CCR5 and ACE2, respectively (ClinicalTrials.gov NCT03164135, NCT04990557).

CRISPR/Cas-based clinical trials have been initiated for the treatment of genetic diseases. The first Cas9-mediated *in vivo* gene therapy trial was initiated by Editas Medicine and Allergan to treat genetic ocular disease LCA10 by eliminating the disease-causing CEP290 mutant (ClinicalTrials.gov NCT03872479). In another CRISPR/Cas-based *in vivo* clinical trial (ClinicalTrials.gov NCT04601051), NTLA-2001 comprised LNP-encapsulated CRISPR/Cas9 elements targeting TTR to treat ATTR amyloidosis by reducing the concentration of TTR in serum. The safety and pharmacodynamic effects of single escalating doses of NTLA-2001 were evaluated in six patients with hereditary ATTR amyloidosis, and the results demonstrated mild adverse events and dose-dependent pharmacodynamics, and a significant reduction in serum TTR protein was observed (170). In addition, a phase I clinical study was initiated by curing rare diseases to assess the safety and efficacy of a CRISPR-based therapy for the treatment of DMD (ClinicalTrials.gov NCT05514249). CRISPR/Cas-based clinical trial for another genetic disease Hereditary Angioedema have also been initiated (ClinicalTrials.gov NCT05120830). Clinical trials to treat metabolic disorder Diabetes Mellitus, Type 1 (T1D) using CRISPR/Cas edited allogeneic pancreatic endoderm cells have been registered (ClinicalTrials.gov NCT05210530, NCT05565248). Several CRISPR/Cas gene therapy companies like Editas Medicine, CRISPR Therapeutics, EdiGene, and Allife Medical Science and Technology have initiated clinical trials to treat transfusion dependent β-Thalassemia (TDT) (ClinicalTrials.gov NCT05444894, NCT05356195, NCT04925206, NCT03655678, NCT05477563, NCT03728322), and some of these clinical trials have entered phase III. Similar to TDT, CRISPR/Cas gene therapy drugs to treat another severe hematological disease sickle cell disease (SCD) have also been used in clinical trials (ClinicalTrials.gov NCT04774536, NCT05477563, NCT03745287, NCT05329649, NCT04819841) CRISPR/Cas-based gene therapy for IEIs has not yet reached clinical trials and preclinical studies involving CRISPR/Cas-edited human HSCs engrafted into mice to correct mutations of IEIs indicated safety and efficacy. With the development of safer and more advanced gene editing tools, as well as versatile delivery methods, an increasing number of CRISPR/Cas-based clinical trials will be initiated for incurable disorders.

## 4 Conclusions and perspectives

The positive results of clinical trials of viral-based gene addition therapy suggest that autologous HSC gene therapy could provide sustained clinical benefits in patients with IEIs. However, gene addition approaches are not amenable for cases where the physiological regulation of genes is required to avoid transgene-related toxicity and genes with large sizes are difficult to deliver. The CRISPR/Cas gene editing system enables permanent, precise, and flexible gene editing without the drawbacks of semi-random genomic insertion, thus emerging as an important new tool for genetic manipulation of HSCs (28). Advances in CRISPR/Cas gene-editing technology have led to new therapeutic options for a wide range of genetic and nongenetic diseases. CRISPR/Cas-mediated HDR has been applied in the gene correction of some IEIs, including X-SCID, IPEX, X-CGD, and WAS (216, 225, 229, 231).

Although clinical trials of CRISPR/Cas gene therapy are in progress, their application for IEIs is still in its infancy, and many issues still need to be addressed to enable safe and effective clinical application. A critical limitation of the gene therapy approach for IEIs is the broad spectrum of disease categories as well as the varying mutations, which require the development of personalized medicine approaches depending on the specific mutation a patient carries (250). To ensure successful clinical outcomes for gene therapy of IEIs, a sufficient number of gene-corrected HSCs with engraftment capabilities following CRISPR/Cas9 gene editing is of paramount importance (251). HDR occurs most efficiently in dividing cells, and the low efficiency of HDR in HSCs can be improved by manipulating critical factors of DNA repair mechanisms to inhibit the NHEJ pathway (72, 73). Optimizing CRISPR/Cas genome editing tools to maximize editing efficiency while minimizing off-target effects presents directions for advancing genome editing agents (247). The engraftment capability and self-renewal ability of gene-corrected HSCs are critical factors that should be taken into account because manipulating and expanding gene-corrected cells *in vitro* increases the risk of losing the multilineage potential of HSCs (251). Exposure to nucleases and the toxic effects caused by condition agents largely affect the self-renew ability of gene corrected HSCs, implementation of “hit-and-run” strategy to reduce nucleases exposure time and development of advanced conditioning regimens could ameliorate these harmfulness to enhance HSC self-renewal ability (14, 251).

The advent and evolution of BE and PE variants with efficient and precise editing holds tremendous promise for the development of novel gene therapies for IEIs. Moreover, the implementation of decentralized manufacturing will enable the translation of gene-modified cell therapies from basic research to hospital-based clinical trials.

## Author contributions

XL, GL, XH designed the manuscript, XL drafted the manuscript, XL, YL prepared figures for the manuscript, FZ, GL, KL, and XH reviewed and revised the manuscript. All authors contributed to the article and approved the submitted version.
